# Toward a Modern View of Pavlovian Conditioning in Applied Behavior Analysis

**DOI:** 10.1007/s40614-026-00494-4

**Published:** 2026-02-20

**Authors:** Matthew Lewon, Michael Domjan

**Affiliations:** 1 University of Nevada, Reno, Psychology Department, MS 296, 1664 North Virginia Street, Reno, NV 89557 USA; 2 University of Texas, Austin, TX USA

**Keywords:** Pavlovian conditioning, Respondent conditioning, Operant conditioning, Pavlovian-operant interactions, Applied behavior analysis

## Abstract

The characterization of Pavlovian conditioning in behavior analysis is outdated. Pavlovian conditioning is not limited to the conditioning of glandular and visceral responses; it also involves responses that participate in operant contingencies, and the elicitation of motivational or emotional states that affect operant behavior. Contemporary formulations emphasize the inseparability of behavior from the antecedent contextual stimuli in the presence of which the behavior occurs. Operant procedures also involve various relations between behavior and antecedent and consequential stimuli, making Pavlovian relations an integral but often overlooked part of operant procedures. Considering these issues encourages increased attention to the relationship of behavior to antecedent context and interactions between Pavlovian and operant relations in applied behavior analysis procedures. We argue that adopting a modern view of Pavlovian conditioning should increase the analytic tools available to behavior analysts, and to the development of new and more effective treatment strategies in the domains of maintenance/generalization of behavior change and behavioral assessment.

## Introduction

B. F. Skinner introduced Pavlovian conditioning in his book *Science and Human Behavior* by recounting a story that Bernard Shaw had written to mock the work of Pavlov (Skinner, 1953). In this story Bernard Shaw characterized salivary conditioning as just concerned with “the spittle” of dogs and so obvious that it was something “known to every child.” Skinner was not as dismissive of Pavlov’s research as was Shaw, but Skinner considered Pavlov’s work to be of limited interest in the quest to create a science of human behavior. He noted that “... if we were to assemble all the behavior which falls into the pattern of the simple reflex, we would have only a very small fraction of the total behavior of the organism” (p. 49).

For Skinner, Pavlovian conditioning was primarily involved in the modification of responses that involved smooth muscles which are “particularly concerned with the internal economy of the organism” (Skinner, 1953, p. 49). Skinner also considered Pavlovian conditioning to be “a process of stimulus substitution” whereby “a previously neutral stimulus acquires the power to elicit a response which was originally elicited by another stimulus” (p. 53). Skinner noted that stimulus substitution enables new stimuli to elicit a response but does not allow the learning of new responses.

Skinner’s characterization of Pavlovian conditioning has turned out to be largely incomplete. For example, Pavlovian conditioning is no longer considered to be primarily “a process of stimulus substitution” (Urcelay & Domjan, 2022). It should be no surprise that our understanding of Pavlovian conditioning, operant conditioning, and their relations has changed significantly in the more than 70 years since *Science and Human Behavior* was published. That is the measure of progress in science. However, Skinner’s views of Pavlovian conditioning remain important because they continue to influence how Pavlovian conditioning is regarded in applications of behavior analysis. For example, in their encyclopedic book on applied behavior analysis (ABA), which runs nearly 900 pages, Cooper et al. (2020) devoted fewer than 2 pages to a general description of Pavlovian or respondent conditioning. Echoing Skinner (1953), they ended the section by noting that “Respondents make up a small percentage of the behaviors typically of interest to applied behavior analysts” (Cooper et al., 2020, p. 32).

Skinner was primarily concerned with how new responses are learned and how organisms interact with and alter their external world rather than their “internal economy.” He noted that “we are most often interested... in behavior which has some effect on the surrounding world. Such behavior raises most of the practical problems in human affairs” (Skinner, 1953, p. 59). Because ABA focuses on “practical problems in human affairs,” operant rather than Pavlovian conditioning became the primary technique used in applications of conditioning principles.

We are not here to argue that operant conditioning was the wrong procedure to focus on in efforts to apply laboratory findings to the solution of behavioral issues of social significance. Obviously that choice has worked out well and has been the foundation of the large and highly successful field of ABA. Unlike some other authors (e.g., Donohoe & Vegas, 2021), we are also not here to provide a detailed summary of new perspectives on Pavlovian conditioning for the benefit of the ABA community. Rather, our goal is to question two key assumptions that have encouraged a deemphasis on Pavlovian conditioning in favor of operant procedures in ABA. The first assumption we will challenge is that Pavlovian conditioning is primarily involved in the modifications of glandular and physiological responses that have little to do with how organisms interact with and manipulate objects in their environment. The second assumption we will dispute is that operant and Pavlovian conditioning are independent forms of learning and therefore one can pursue a science of operant behavior without paying much attention to Pavlovian processes.

In developing our argument, we begin with a discussion of the history of Pavlovian conditioning in ABA. We then discuss Pavlovian conditioning of lever pressing in rats and key pecking in pigeons. We focus on these two examples because they are paradigmatic operant responses, employed in much of the laboratory research on operant conditioning. We then examine the assumption that one can study operant learning without regard to possible interactions with Pavlovian processes. We will argue that Pavlovian and operant learning are inextricably intertwined. This raises the possibility that Pavlovian procedures may be used to control operant behavior. The validity of that prediction has been confirmed by a great deal of experimental evidence. We review that evidence and then end the article with a discussion of possible ways in which a contemporary perspective on Pavlovian conditioning could expand the analytic tools and procedures available in ABA research and practice.

## Historical Context for Pavlovian Conditioning in ABA

American perspectives have dominated the development of modern psychology (Thalmayer et al., 2021) and these (especially behavioristic approaches) have been characterized by pragmatism, or the assignment of value to theories or approaches to psychological phenomena based on the extent to which they contribute to the solution of problems of social concern (Hayes & Fryling, 2019; Hayes et al., 1988; James, 1907; Leão et al., 2016; Skinner, 1953). This is exemplified by John B. Watson’s presidential “behaviorist manifesto” address to the American Psychological Association in which he stated that the goal of psychology (and therefore the criterion by which theories/approaches to psychology ought to be evaluated) is the prediction and control of behavior (Watson, 1913, p. 158).

Around the time of Watson’s address, translations of I. P. Pavlov’s research were becoming available in English (Pavlov, 1906; Yerkes & Morgulis, 1909). It is interesting to note that although Pavlov studied the effects of arranging various relations between stimuli on learning and behavior, he was primarily interested in what this could reveal about the nervous system (Pavlov, 1932). On the other hand, Watson saw the practical implications of this work and the potential application of it to psychological problems. Watson and Rayner’s (1920) Little Albert study, in which they reported the establishment of a conditioned fear response in a young child with a procedure similar to that described by Pavlov, is an early example of translational research aimed at validating laboratory findings through demonstration of similar phenomena in a more natural setting.

Watson’s student Mary Cover Jones (1924a, 1924b) took this a step further by using exposure procedures similar to Pavlovian extinction procedures to alleviate conditioned fear responses in children (Alpers, 2024). This work evolved into what would become known as systematic desensitization (Wolpe, 1958) and various other “exposure” and “counterconditioning” therapeutic applications in clinical psychology. These therapeutic applications led to the establishment of the field known as *behavior therapy* (Alpers, 2024; Barlow, 1997; Thomas & O’Callaghan, 1981). Features of exposure and counterconditioning derived from Pavlovian conditioning principles are also prominent in acceptance and commitment training (ACT), a contemporary behavioristic approach to managing troublesome thoughts and emotions arising from language (Hayes et al., 2012).

What is now known as ABA (Cooper et al., 2020) arose from a similar early 20th-century context of American pragmatism, behaviorism, and a desire to apply knowledge derived from psychological laboratory research to issues of social concern. ABA is acknowledged to have its origin in the conceptual work of B. F. Skinner and followers in his intellectual tradition (Michael, 1980; Morris et al., 2005). Like Watson, Skinner asserted that the value of psychology as a science was in its use in solving problems and its goal is the prediction and control of behavior (Skinner, 1953, 1971). However, unlike Watson and as noted above, Skinner viewed Pavlovian conditioning as both relatively insignificant and inadequate for the task of solving significant human problems.

Jack Michael (1980) credited Skinner’s (1935, 1937, 1938) construction of a dichotomy between Pavlovian/respondent and operant conditioning processes as a major contribution to the development of the field:

Prior to 1930 there wasn’t much going on. Of course, Watson and others had begun the behavioral movement, but *the critical distinction between operant and respondent relations was lacking and everyone was trying to interpret operant processes and relations in terms of respondent conditioning, and not getting anywhere*. In the period of 1930 to 1938, Skinner put it all together. He managed, within a brief but fertile period, to come up with almost all of the essential methods, concepts, and functional relations of our field as we see it today: a focus on the behavior of the single organism, rate of response as the main dependent variable, the cumulative record, *the operant-respondent distinction with the related difference between the CS and the S*^*D*^, and the various kinds of intermittent reinforcement. (Michael, 1980, p. 1; emphasis added)

Skinner and his followers’ conceptual views of Pavlovian/respondent conditioning, along with emerging demonstrations of the success of applications based on operant procedures in the 1950 s and 1960 s (e.g., shaping and other forms of differential reinforcement, operant extinction, and punishment procedures) with populations for whom other approaches were relatively ineffective (e.g., individuals with autism and intellectual and developmental disabilities), undoubtedly contributed significantly to the emphasis on operant conditioning in the development of ABA.

Other factors that contributed to the predominance of operant conditioning procedures in ABA included the founding of the *Journal of the Experimental Analysis of Behavior* (*JEAB*) in 1958. *JEAB* provided a forum for operant-focused researchers to share their empirical and conceptual work (Catania, 2008), but it has been suggested that this also contributed to a prevailing divide between research conducted from a Skinnerian operant-focused perspective and research focused on Pavlovian conditioning and its relation to operant conditioning (Williams, 1987). It is possible that this segregation is responsible in part for the proliferation of outdated Skinnerian views regarding Pavlovian conditioning (Domjan, 2016) and outdated views of the relevance of Pavlovian conditioning to ABA (discussed below).

Another factor may have been the different contexts in which procedures derived from basic Pavlovian and operant research were being applied. At the time of the earliest ABA studies (late 1950 s through the 1960 s), applications of exposure-based Pavlovian conditioning principles were largely confined to outpatient psychotherapeutic settings, with little contact with recipients of psychological services outside of clinics (Barlow, 1997; Benjamin, 2005). Because of this, behavior therapeutic technologies derived from basic Pavlovian conditioning became the professional domain of clinical psychologists. In contrast, early ABA research and practice occurred in settings that afforded more extensive observations and greater influence over individuals’ environments: psychiatric facilities, institutions for the intellectually and developmentally disabled, schools, and home settings. This contrast may have contributed to a division between the development of applications of Pavlovian and operant conditioning, either related to issues of which professionals had domain over each, or to perceptions of Pavlovian and operant procedures as only being relevant to certain populations or therapeutic problems.

By the late 1960 s, enough proto-ABA research was being conducted to support the establishment of the *Journal of Applied Behavior Analysis* (*JABA*) as a counterpart to the predominantly basic research published in *JEAB*. In the first issue of *JABA*, Baer et al. (1968) provided an influential description of what they considered to be the defining features of ABA, an article that continues to be a part of the foundational knowledge for individuals seeking certification from the Behavior Analyst Certification Board (BACB, 2017, item A-5). Although Baer et al. were writing from a Skinnerian/operant perspective, there is nothing in their article prescribing that ABA be limited to practices explicitly derived from operant conceptualizations and procedures, nor that ABA be limited to applications in certain settings or with specific populations. Rather, they suggested that ABA should be defined by broader characteristics, including a focus on socially significant behavior change and systematic demonstrations of the efficacy and replicability of the procedures. As such, at this stage in the development of ABA, anybody (e.g., researchers, clinical psychologists, counselors, educators) could be considered to be conducting ABA if their work exhibited the dimensions Baer et al. described.

The broad conceptual approach of Baer et al. (1968) began to change with the professionalization of ABA through the accreditation of ABA training programs and the certification and licensure of individual ABA practitioners. As has happened in related fields (e.g., clinical psychology; Benjamin, 2005), this involved defining who is allowed to practice, the settings in which they are to be trained, the nature of that training (e.g., areas for didactic and experiential training), the practices that are considered within the scope of the profession, and the populations with whom they may work.

A particularly influential development in the professionalization of ABA has been the BACB’s *Board Certified Behavior Analyst (BCBA)/Board Certified Assistant Behavior Analyst (BCaBA) Task List* (e.g., BACB, 2012, 2017), which is now in its sixth edition and is called the *BCBA Test Content Outline* (BACB, 2022). This serves as the basis for the contents of the BCBA and BCaBA certification exams. It is also often used as a framework for the supervised fieldwork experience hours, which are a requirement to sit for the exam (BACB, 2024). Due to its importance in certification, some ABA-focused textbooks (e.g., Cooper et al., 2020; Malott & Kohler, 2021) have explicitly tied their content to task lists. They are, therefore, one indicator of the perceived relevance of Pavlovian conditioning to ABA. The *5th Edition Task List* (BACB, 2017), contained 95 items, and only 1 of these, in the Concepts and Principles section, directly related to Pavlovian conditioning: “B-3 Define and provide examples of respondent and operant conditioning.” The current *BCBA Test Content Outline* (BACB, 2022) contains 104 items, with just 1 directly related to Pavlovian conditioning: “B.3. Identify and distinguish between respondent and operant conditioning.”

Another indicator of the status of Pavlovian conditioning within ABA, both in terms of its perceived importance and in providing an up-to-date overview of what is known about it, is its representation in textbooks. Madden et al. (2023) reviewed several ABA-focused textbooks and concluded that Pavlovian conditioning continues to be characterized largely in terms of temporal contiguity or “pairing” of stimuli, a view that is overly simplistic in light of contemporary research (Rescorla, 1988). All the texts reviewed by Madden et al. devote space to the distinction between operant and Pavlovian conditioning. The coverage of applications derived from Pavlovian conditioning research varies across texts but is typically small compared to the coverage of procedures considered to be operant in nature. Topics in which Pavlovian conditioning is explicitly discussed in terms of application include the establishment of conditioned reinforcers, escape and avoidance behavior, and systematic desensitization and related exposure procedures for fear or anxiety. The textbook by Martin and Pear (2019) is notable for including a chapter dedicated to interactions between operant and Pavlovian conditioning in applied settings.

As we pointed out, diverse historical factors have contributed to the current status of Pavlovian conditioning in contemporary ABA training and practice. The result has been that (1) representation of Pavlovian conditioning in ABA is outdated and overly simplistic; and (2) the relevance of Pavlovian conditioning to applied matters and operant conditioning procedures is not fully appreciated. In the following sections, we review phenomena that encourage a more integrated approach that recognizes that operant conditioning inevitably involves Pavlovian components. We then argue that recognizing these Pavlovian components provides the applied behavior analyst with additional tools to change operant behavior.

## Pavlovian Conditioning of Paradigmatic “Operant” Responses

In pursuing the development of the science of operant conditioning, Skinner and his associates concentrated on two skeletal responses: pigeons pecking an illuminated response key and rats pressing a response lever near a food cup. As it turns out, both of these responses can be conditioned with Pavlovian, rather than operant, procedures. Furthermore, these are just two of many examples of the Pavlovian conditioning of skeletal responses (e.g., Jenkins et al., 1978; Staddon & Simmelhag, 1971; Timberlake & Grant, 1975; Timberlake et al., 1982). Appetitive Pavlovian conditioning often results in approaching and manipulating the conditioned stimulus (CS). If the unconditioned stimulus (US) is a sexual reinforcer, the conditioned response may also involve attempts to copulate with the CS (Köksal et al., 2021). Describing all of these examples would take us too far afield, but given the central role of key pecking and lever pressing in analyses of operant behavior, we provide more details about the Pavlovian conditioning of those responses.

### Pavlovian Conditioning of Key Pecking in Pigeons

Studies of the Pavlovian conditioning of key pecking in pigeons started with the discovery of the phenomenon of “autoshaping” by Brown and Jenkins (1968) 15 years after the publication of Skinner’s *Science and Human Behavior*. The authors tested procedures in which presentation of a key light ended in delivery of grain to a feeder below the pecking key. Pecking the key light was not required for the delivery of grain, making this a Pavlovian conditioning procedure.

The original goal of these experiments was to see if pigeons would learn to peck the key light without the use of a shaping procedure that was routinely employed at that time as a preliminary training phase in studies of operant pecking behavior. Brown and Jenkins (1968) conducted two experimental sessions of 80 trials each. Of the 70 pigeons they tested, 68 birds pecked the key light at some point during the 160 conditioning trials. For most of the birds, the first peck occurred between trials 20 and 40. Pecking did not reliably occur in various control groups that did not involve forward pairings of the key light with food. The emergence of pecking without a shaping procedure or the requirement that the pigeons peck the key to activate the food magazine was a striking finding given that pecking was viewed as a paradigmatic example of operant behavior.

Critical to a Pavlovian interpretation is ruling out the possible role of operant reinforcement of pecking. The presentation of grain could accidentally reinforce the pecking response even if pecking was not required to produce the food. However, the first peck at the key-light could not be explained by adventitious reinforcement because such reinforcement could occur only after the first peck was performed. For that reason, Brown and Jenkins (1968) focused on when the pigeons made their first peck. Forward pairings of the key-light with food facilitated the occurrence of the first peck in comparison to various control procedures such as reverse pairing of food delivery and illuminated key-light and unpaired illumination of the key-light and food delivery.

Another strategy for ruling out the role of adventitious operant reinforcement is the use of the omission control procedure. The omission control was first developed to study the possible role of operant reinforcement in salivary conditioning in dogs (Sheffield, 1965). The procedure called for omitting the food US if the dog made a salivary conditioned response (CR) on a particular trial. Use of this procedure did not prevent the development of conditioned salivation. Sheffield reported that one of his dogs, Vicki, persisted in conditioned salivation during the course of 800 omission control trials.

The omission control was introduced to research on the Pavlovian conditioning of key pecking by Williams and Williams (1969; see also Hershberger, 1986). In their experiment, a 6-s key light ended with access to grain provided that the bird did not peck the key light. If a peck occurred during the key light, the grain was cancelled on that trial. For 10 of the birds, this omission procedure was in effect from the start of parings of the key light with food. Because pecks canceled food delivery for these birds, pecking the key light could not have been reinforced by the grain presentation. Although there was considerable variability, all 10 birds showed acquisition of the pecking response, which persisted for all but 1 pigeon. For birds whose pecking was initially reinforced, shifting them to the omission procedure slightly decreased rates of responding, but responding continued across over 1,000 trials. Thus, the Pavlovian contingency between the key light and food was sufficient for both acquisition and maintenance of key pecking.

The pioneering experiments by Brown and Jenkins (1968) and Williams and Williams (1969) were provocative and stimulated a great deal of subsequent research. In summarizing this research, Schwartz and Gamzu (1977/2022, p. 60) wrote, “The data that have been presented here overwhelmingly suggest that autoshaping is Pavlovian.” This line of research left little doubt that the acquisition of key pecking in pigeons is not limited to operant procedures and provided a dramatic counterexample to the claim that Pavlovian conditioning is primarily concerned with the conditioning of glandular responses.

### Pavlovian Conditioning of Lever-Pressing in Rats

Soon after the discovery of Pavlovian conditioning of the pigeon’s key-peck, investigators developed procedures for Pavlovian conditioning of lever-contact and lever-press responses in rats. The response levers used in these experiments were retractable and could be mechanically extended or withdrawn from the experimental chamber. In addition, the levers were illuminated when they were extended into the chamber. At the start of a conditioning trial, a lever was lit and extended into the experimental chamber, and then withdrawn when the reinforcer was delivered. Thus, the extension of the lever served as the CS.

The first experiment on “autoshaping” of lever press behavior in rats was conducted by Peterson et al. (1972). A food cup was located on the front wall of a small experimental chamber, and retractable and illuminable levers were mounted on each of the side walls. A conditioning trial started with the extension and illumination of one of the two levers for 15 s. Extensions of the lever on one side wall always ended with food delivery. Extensions of the lever or the other side wall ended without consequence. Contacts with each lever were recorded on each trial. Pairings of the response lever with food resulted in orderly acquisition of lever contact responses, which declined in a subsequent phase when an extinction procedure was introduced. Contacts were significantly lower for the lever that was not paired with food.

When food was the US, lever contacts consisted of licking and gnawing the lever. For comparison, Peterson et al. (1972) also tested a group of rats that received brain stimulation as the US. With rewarding brain stimulation, lever contacts consisted mostly of sniffing and pawing the response lever. Of course, in order to make contact with the response lever, the rats had to first approach the lever on a given trial. Therefore, a conditioned approach response also occurred when the lever was paired with food or brain stimulation. Like the pigeon autoshaping experiments, these results raise questions about the use of lever-directed behavior as a paradigmatic operant rather than Pavlovian response.

In the original studies of Pavlovian lever-directed behavior, the response lever was illuminated when it was extended into the experimental chamber at the start of a trial. In the typical operant lever pressing experiment, the lever used is not illuminated. Experiments by Atnip (1977) showed that illumination of the retractable lever is not necessary for lever-contact responses to develop with a Pavlovian procedure. Atnip (1977) also reported acquisition of lever-press responses when an omission control procedure was used with a nonilluminated lever. Pavlovian conditioning of lever contact responses in rats using an omission control procedure continues to be a topic of contemporary interest (e.g., María-Ríos et al., 2023).

Pavlovian conditioning of approach and contact with a CS can also develop even if the CS is not a retractable lever. Cleland and Davey (1983) compared Pavlovian conditioning of rats with a localized light or a clicker CS paired with food. The rats came to approach the light that was paired with food but did not show this approach response to the clicker. Instead, the clicker paired with food came to elicit approach and exploration of the food cup. Because all of these are skeletal responses, the study by Cleland and Davey provides additional evidence against Skinner’s claim that Pavlovian conditioning is limited mainly to glandular responses. In addition, this experiment illustrates that different forms of skeletal behavior can be conditioned depending on the nature of the CS. Approach to the CS as a CR has come to be known as *sign tracking*, whereas approach to the food cup as a CR has come to be referred to as *goal tracking* (for more recent studies of sign tracking vs. goal tracking, see Burns & Domjan, 2001; Colaizzi et al., 2020; Flagel et al., 2009).

### From Spontaneous Operants to the Three-Term Contingency

As we mentioned earlier, in his effort to argue for a new science of operant behavior, Skinner proposed a distinction between emitted and elicited behavior (Domjan, 2016). He viewed elicited behavior as closely related to reflexes or reactions to a clearly identifiable triggering or eliciting stimulus. Skinner viewed Pavlovian conditioning to be elicited. The new science of operant conditioning was to be a marked departure from reflex systems, involving emitted rather than elicited behavior (Michael, 1980).

The concept of “emitted” behavior was appealing because it represented a major departure from the earlier reflex tradition of Pavlov and seemed to provide more flexibility in how an organism interacted with its environment. In characterizing operant behavior as “emitted,” Skinner noted that “no correlated stimulus can be detected upon occasions when it is observed to occur” (Skinner, 1938, p. 21). The idea that operant behavior occurs in the absence of a clear triggering stimulus was adopted in subsequent landmark volumes defining the new science of operant conditioning. Keller and Schoenfeld (1950), for example, noted that “responses that are *emitted* more or less independently of identifiable stimuli, we may now call *operant”* (p. 49, emphasis in original).

The relative difficulty of identifying current stimuli that contribute to the occurrence of a given instance of behavior may encourage the conclusion that some behaviors occur independent of the influence of the contexts in which they occur or are in some way “spontaneous.” Skinner and others have written inconsistently about this issue. On the one hand, operant behavior has been distinguished from respondent behavior in that the former is held to be *emitted* and probabilistic, but the latter is *elicited* and occurs in an invariant relation with antecedent stimuli (Domjan, 2016; Keller & Schoenfeld, 1950, p. 49; Skinner, 1938, p. 21). On the other hand, as Skinner himself pointed out, “Spontaneity is negative evidence; it points to the weakness of a current scientific explanation, but does not itself prove an alternative version. By its very nature spontaneity must yield ground as a scientific analysis is able to advance” (Skinner, 1953, p. 48). Skinner (1938) noted that operant behavior “is studied as an event appearing *spontaneously* with a given frequency” (p. 21, emphasis added), but earlier on the same page he wrote that “an operant may and usually does acquire a relation to prior stimulation.” Likewise, Keller and Schoenfeld (1950) noted, “Operant behavior, however, *spontaneous* in its initial occurrence, very soon becomes associated with stimuli” (p. 52, emphasis added). In a similar vein, Ferster and Skinner (1957) commented, “The effect of reinforcement is maximally felt when precisely the same conditions prevail” (p. 9). Thus, spontaneity in historical characterizations of operant behavior has gone hand-in-hand with the recognition that operant responses are closely related to antecedent stimuli.

The importance of antecedent stimuli in operant conditioning was first recognized by Thorndike (1911). Thorndike is frequently acknowledged to have initiated the study of instrumental or operant conditioning with his puzzle box experiments (Chance, 1999). Thorndike placed young cats in small boxes with various types of latches. The cat could escape from a box and obtain a piece of food if it manipulated the escape latch in the required manner. Different boxes required different responses to release the escape door. The response required in a particular box became more likely with repeated training trials. Thorndike summarized these results by noting that a response became more likely if the performance of that response resulted in a “satisfying” state of affairs.

Skinner characterized the discovery of increased efficiency of escaping from a puzzle box as Thorndike’s Law of Effect (e.g., Skinner, 1953, p. 60) and noted that “The Law of Effect is not a theory” (Skinner, 1953, p. 81). We agree that describing the outcome of the puzzle box experiments is not a theory but an empirical generalization. But Thorndike did not offer such an empirical generalization as the Law of Effect (Domjan, 2026). Rather, Thorndike’s statement of the Law of Effect is that.

... of several responses made to the same situation, those which are accompanied or closely followed by satisfaction to the animal will, other things being equal, be more firmly connected to the situation, so that, when it recurs, they will be more likely to recur. (Thorndike, 1911, p. 244)

What makes this a theoretical claim rather than an empirical generalization is the phrase “more firmly connected to the situation.” In more modern terminology, we use S to designate the situation or stimuli in the presence of which a response occurs, R to represent the response, and O to represent the response outcome (or the “satisfaction” or reinforcer produced by the response in Thorndike’s terms). Thorndike’s statement of the Law of Effect claims that an operant conditioning procedure results in the formation of an S-R association.

In describing the Law of Effect, Skinner ignored Thorndike’s emphasis on the learning of an S-R association. But neither Skinner nor subsequent behavior analysts were able to escape the fact that the specification of an operant response R inevitably occurs in the presence of specific cues (S) experienced at the time the response is performed. For example, the specification of a pigeon’s key-peck response is inevitably accompanied by a restricted set of visual and spatial stimuli related to the response key. Likewise, the specification of a lever press response automatically restricts the set of visual, spatial, and tactile cues the rat encounters when it presses the bar. In an effort to remove distinctive stimuli associated with different responses, some investigators arranged for rats to push a vertical rod either to the right or the left as an instrumental response in the same experimental chamber (e.g., Colwill & Rescorla, 1986). However, one can argue that even this special arrangement did not eliminate different cues being associated with each response because pushing the rod to the left or the right exposed rats to different spatial cues. Thus, the generic operant conditioning procedure is not correctly characterized as involving just a response R that results in the delivery of a reinforcer O. Rather, the fundamental operant conditioning procedure must also include the unique stimuli S in the presence of which the response is performed.

Recognition that there are three basic elements to an operant conditioning procedure (S, R, and O, or antecedent, behavior, and consequence; A, B, C) became widely accepted among behavior analysts (Ferster & Skinner, 1957; Keller & Schoenfeld, 1950; Michael, 2004) and continues to be the primary analytic framework in ABA (Cooper et al., 2020). The relations between these three elements were described as the three-term contingency. In Skinner’s own words:

Any stimulus arising from the space, the operandum, or special stimulating devices prior to the response is designated as S^D^. A response, such as pressing the lever or pressing the disc is R. Food presented to a hungry organism is a positive reinforcer (S^reinf^), a bright light or shock a negative reinforcer. The interrelations among S^D^, R, and S^reinf^ compose the contingencies of reinforcement. All three terms must be specified. (Skinner, 1969, p. 23)

As one of us has argued elsewhere (Domjan, 2016), a commitment to the three-term contingency is a rejection of the idea that operant behavior occurs spontaneously or is “emitted” rather than evoked or elicited. We discuss the implications of this for ABA in greater detail later in the article. However, the historical development of the three-term contingency also has implications for the Skinnerian dichotomization of Pavlovian and operant conditioning as separate and independent forms of learning. We turn to that topic next.

### Why Operant Conditioning Cannot Be Divorced from Pavlovian Conditioning

One of the major reasons why Skinner did not take Pavlovian conditioning more seriously is that he believed that Pavlovian and operant conditioning were independent processes. If one makes that assumption, one can pursue a science of operant conditioning without taking Pavlovian learning into account. As Skinner put it, in a Pavlovian procedure, “a reinforcer is paired with a stimulus; whereas in operant behavior it is contingent on a response. Operant reinforcement is therefore a separate process and requires a separate analysis” (Skinner, 1953, p. 65). In discussing these two forms of learning, Skinner elevated procedural differences between Pavlovian and operant conditioning to the status of differences in the underlying learning processes that are involved.

One can make a strong argument that Pavlovian conditioning can occur independently of operant learning on the basis of the omission control procedure. As we noted earlier, Pavlovian CRs can develop even if such responses cancel the delivery of the US and are therefore never paired with that US or reinforcer. However, the omission control procedure could not have been the basis for Skinner’s claim that Pavlovian and operant conditioning are independent of each other because the omission control procedure was developed more than a decade after Skinner made that assertion.

Given the results of numerous studies using the omission control procedure, Pavlovian learning independent of operant learning is now well-established. Is the reverse also true? That is, can operant learning occur in the absence of Pavlovian conditioning? That is much more difficult, if not impossible, to prove. As we have pointed out, analyses of operant conditioning dating back to Thorndike have emphasized that the essential features of operant conditioning include three terms: S, R, and O. This sequence of events allows for stimulus S to be paired with O. These pairings can result in an S–O association, which is a form of Pavlovian conditioning. One cannot prevent the formation of an S–O association without breaking up the three essential elements of an operant procedure. This line of reasoning suggests that it is not possible to design an operant analogue of the omission control procedure. The S-R-O sequence that characterizes operant procedures always permits the pairings of S with O, and consequent Pavlovian learning. Given that S–O associations are always possible in an operant procedure, the independence of operant and Pavlovian learning is difficult to demonstrate empirically.

If S–O pairings and Pavlovian learning cannot be eliminated from operant procedures, viewing operant and Pavlovian learning as independent is not a viable path forward. Rather, one must face the question of how operant and Pavlovian learning may interact to generate the behavioral changes produced by operant procedures. That research question has not attracted much attention among investigators working in the Skinnerian tradition but has been the focus of a great deal of research and theoretical speculation among scientists pursuing more traditional approaches to the study of learning.

### Interactions between Pavlovian and Operant Conditioning

The interaction of Pavlovian learning with operant contingencies was at the heart of the two-factor theory of avoidance learning, which was introduced in the middle of the twentieth century (Mowrer, 1947). According to this theory, the acquisition of avoidance behavior first requires the Pavlovian conditioning of fear to stimuli that predict danger. In the presence of fear, operant responses can be negatively reinforced by fear reduction, which, in turn, leads to the acquisition of avoidance responding. These basic ideas continue to guide contemporary analyses of avoidance learning (LeDoux et al., 2017).

Another early theory of the interaction of Pavlovian and operant conditioning was proposed by Hull (1930, 1931) and adopted by Spence (1956) and others. According to this theory, an instrumental or operant procedure leads to the Pavlovian conditioning of an anticipatory goal response or reward expectancy. This Pavlovian CR is assumed to become a cue for the operant response through the operations of Thorndike’s S-R learning mechanism. In more contemporary terminology, operant conditioning procedures result in the Pavlovian learning of a reward expectancy. That Pavlovian reward expectancy then motivates the operant response.

### Concurrent Measurement of Pavlovian and Operant Responses

Early formulations of how Pavlovian learning may be involved in the learning of instrumental or operant behavior stimulated numerous experiments, which were reviewed in a landmark article by Rescorla and Solomon (1967). One line of inquiry, referred to as concurrent measurement experiments, involved the measurement of Pavlovian conditioned responses during operant conditioning procedures. The idea was that if Pavlovian CRs provided the impetus for operant behavior (as postulated by Hull and Spence), one should be able to observe the Pavlovian CR as an antecedent to the operant response in operant conditioning procedures. Experimental tests of this hypothesis provided mixed results.

In studies of avoidance conditioning, concurrent measurement experiments have shown that Pavlovian conditioned fear dissipates with extended training while avoidance behavior persists (Kamin et al., 1963; Mineka, 1979). In operant conditioning with food as the reinforcer, investigators have observed the development of conditioned salivation. However, the Pavlovian salivary response can occur before, along with, or after the operant response (e.g., Ellison & Konorski, 1964; Williams, 1965).

Evidence of dissociations between a Pavlovian CR and operant behavior may reflect the independence of Pavlovian and operant conditioning. However, one can also argue that the Pavlovian CR measured in a particular experiment was not the correct one to illustrate how Pavlovian conditioning mediates operant learning. In appetitive conditioning with food, for example, Pavlovian CRs can include not just salivation but also other physiological changes as well as sign tracking or goal tracking. One of these responses may be more important in mediating operant behavior than another. This argument, unfortunately, is not very helpful because there is no theoretical basis for selecting one Pavlovian CR as more important in the mediation of operant behavior than another.

### The Pavlovian-Instrumental Transfer Design

The failure of concurrent measurement experiments to elucidate the relationship between Pavlovian and operant learning led to the abandonment of that strategy and its theoretical foundation. As an alternative, Rescorla and Solomon (1967) suggested that the critical outcome of Pavlovian conditioning is not the conditioning of a specific CR elicited by the CS but the activation of an emotional or motivational state that can influence the performance of an operant response. Terms like “motivational/emotional state” are not presently in the typical vocabulary of behavior analysts, but the use of these terms by Rescorla and Solomon is similar to the language used by Estes and Skinner (1941) to describe conditioned suppression as a result of a conditioned emotional state of “anxiety,” and by Skinner (1953, p. 165) in his analysis of the effects of motivational and emotional states on operant behavior (Lewon & Hayes, 2014).

Along with their theoretical proposal, Rescorla and Solomon advocated a new strategy for investigating the relationship between Pavlovian and operant conditioning. This strategy stems from the key implication of their theory: “Pavlovian conditioning procedures should have important effects upon instrumental behavior” (Rescorla & Solomon, 1967, p. 170). Furthermore, these effects should be evident when a Pavlovian CS is presented during operant responding “in the absence of changes in instrumental contingencies” (Rescorla & Solomon, 1967, p. 170). The technique Rescorla and Solomon advocated has come to be known as the *Pavlovian-instrumental transfer* (PIT) design.

The PIT design does not make any ambitious claims about the role of Pavlovian mechanisms in the learning of an operant response. Rather, it is based on the simple proposition that Pavlovian CSs can influence the performance or rate of operant behavior. The details of these influences are studied using an experimental design that arranges both Pavlovian and operant relations, either concurrently or in succession, and observing how the effects of these interact. One form of training is devoted to Pavlovian conditioning of a CS. The other form of training involves conditioning of an operant response. A subsequent test phase is devoted to observing how the rate of the operant response changes when the Pavlovian CS is presented.

A study conducted by Estes and Skinner (1941) may have been the first experiment using the PIT design (before the term “PIT” was used). Rats’ lever presses were reinforced with food on a variable-interval schedule. During the lever press training sessions, a tone was presented occasionally, each time ending in a brief shock. After just a few tone-shock pairings, presentation of the tone resulted in near total suppression of lever pressing, which resumed after the tone ended. Thus, a Pavlovian conditioned aversive stimulus significantly disrupted operant lever pressing reinforced with food, even though the schedule of food reinforcement was unchanged. This experiment was the first demonstration of what has come to be known as the conditioned suppression effect. Subsequent research indicated that the degree of conditioned suppression depends on features of both the Pavlovian and operant procedures that are used (see reviews by Blackman, 1977/2022; Lyons, 1968).

In the Estes and Skinner experiment, the three components of the PIT design were conducted in the same experimental sessions. Subsequent PIT experiments involved greater separation of these three components. For example, another early PIT experiment involved testing the effects of an appetitive Pavlovian CS on appetitive operant conditioning (Estes, 1948). Rats first contacted Pavlovian conditioning in which a tone was paired with the presentation of food in a chamber that lacked a response lever. In the next phase, a response lever was extended into the chamber and lever pressing was reinforced with food. This phase was followed by the transfer test during which the response lever was available and the tone was presented periodically. As is common in contemporary research, the transfer test was conducted in extinction with food pellets no longer delivered. In this transfer test, presentation of the Pavlovian CS increased operant lever pressing.

These studies represent just two of many different types of PIT studies. The operant response can be maintained by positive or negative reinforcement, and these procedures can involve procedures with or without experimenter-defined S^D^ and S^**△**^ stimuli. The Pavlovian CSs presented during operant responding can be conditioned with an appetitive or aversive US, and can involve either excitatory (CS +) or inhibitory (CS–) conditioning. These variations result in 16 different types of PIT experiments, many of which have been investigated (see Table 1 in Rescorla & Solomon, 1967). PIT experiments can also involve other variations in the operant procedures (different schedules and delays to reinforcement) as well as changes in the Pavlovian procedures (e.g., the CS-US interval). Considering all of these procedural possibilities, the range of potential PIT experimental designs far exceeds the original 16. It is no wonder that the Rescorla and Solomon review generated a great deal of experimental attention (e.g., Overmier & Lawry, 1979; Trapold & Overmier, 1972).

Recent years have seen a major revival of interest in PIT research with both humans and nonhumans (e.g., Badioli et al., 2024; Corbit & Balleine, 2016; Degni et al., 2022; Mahlberg et al., 2021). Much of this renewed interest is encouraged by efforts to better understand the role of incentive motivation and reward expectancy in goal-directed behavior. Incentive motivation and reward expectancy are manipulated using the Pavlovian phase of the PIT design and the effects of those variables are examined in the transfer phase when the Pavlovian CS is presented when the participant has the opportunity to perform the “goal-directed” operant response.

Much of contemporary PIT research has focused on how an appetitive Pavlovian CS (or CS +) alters the rate of a positively reinforced operant response. Two types of transfer effects have been identified, specific transfer and general transfer (Cartoni et al., 2016). A transfer effect is said to be specific if the Pavlovian CS increases only those operant responses that are reinforced by the same reinforcer as the US used in the Pavlovian conditioning phase. A general transfer effect is said to occur if the presentation of *any* appetitive CS increases the rate of behavior on an operant schedule of positive reinforcement.

It has been suggested that whether transfer effects are specific or general depends on whether the experimental procedures encourage distinguishing between different USs or reinforcers (Holland, 2004). Another important factor is the possible interaction between responses elicited by a Pavlovian CS and the operant response required to obtain the reinforcer. As we discussed earlier, a Pavlovian procedure can result in various skeletal CRs, including sign tracking and goal tracking. These CRs can move participants towards or away from an operant manipulandum and influence the outcome of PIT tests (e.g., Holmes et al., 2010; Krank et al., 2008). Consideration of specific skeletal Pavlovian conditioned responses may be particularly important when the PIT design is used in ABA.

## Beyond the Operant-Respondent Distinction: Expanding the Analytic Toolbox in ABA

As we noted at the outset, there is nothing wrong with an emphasis on operant conditioning in ABA; this has been successful in many ways. At the same time, Pavlovian processes are critically involved in what applied behavior analysts are doing in research and service delivery. Explicitly acknowledging this in training and practice may help behavior analysts improve some of their current procedures and create novel solutions to some intractable clinical challenges in ABA practice. We explore these possibilities next.

### Increased Recognition of Antecedent Control of all Behavior

Skinner’s dichotomy between respondent and operant behavior was predicated on the perceived degree of control by antecedent stimuli. Respondent behavior continues to be characterized as having a relatively invariant relation to specific eliciting stimuli. However, research has shown that learning from Pavlovian stimulus–stimulus relations is remarkably sensitive to context beyond an eliciting CS: (1) although the effects of initial learning experiences with CSs in Pavlovian excitatory and inhibitory conditioning procedures generalize readily, subsequent experiences make it such that responses to CSs become increasingly context-specific (Rosas et al., 2013); (2) the topographies of behavior elicited by USs/CSs depend on the contexts in which these occur (Burns & Domjan, 2001; Fanselow et al., 1987); (3) URs/CRs may include “skeletal” responses that participate in operant contingencies (sign tracking, goal tracking, and autoshaping); and (4) topographies of behavior elicited by CSs due to Pavlovian conditioning are often different from those elicited by USs (Timberlake & Grant, 1975).

In contrast to Pavlovian CRs, operant behavior was supposed to be probabilistic, but that view has become difficult to defend. The operant conditioning literature is replete with demonstrations of how the behavioral effects of response–outcome relations (e.g., reinforcement, punishment, and extinction) are limited to contextual circumstances that are similar to those in which those relations occurred (the field of stimulus control; Dinsmoor, 1995; Honig & Urcuioli, 1981). There is also evidence that operant procedures begin to establish stimulus control immediately, even before “intentional” discrimination training is carried out (Dinsmoor, 1995; Dinsmoor et al., 1969). Therefore, features of context may come to control operant behavior before an observer recognizes that this is happening. This may encourage viewing the behavior as occurring “spontaneously,” but that would amount to an imposition of observers’ incomplete perspectives on the events they are observing (Smith, 2007).

As occurs in Pavlovian conditioning (Campolattaro et al., 2008; Dinsmoor, 1995), generalization prevails early in operant training: the effects of the operant response–outcome relation are observed across a relatively wide range of contexts that share some features with the training context. It may seem that the response occurs independent of the context, but the apparent spontaneity arises from a failure to identify all of the contextual factors in the presence of which the response originally occurred, as well as the relation of these to situations in which the response is subsequently observed. As discrimination training proceeds, the features of context that affect behavior gradually narrow to those that are reliably correlated with particular response–outcome relations (e.g., changes in stimulus generalization gradients as a function of discrimination training; Honig & Urcuioli, 1981). As this happens, the control of behavior by specific antecedent stimuli becomes more apparent through systematic changes in some feature of behavior with changes in context.

The main implication of these issues for ABA is that they encourage viewing behavior as continuously and inseparably interrelated with the contexts in which the behavior occurs. Our contention is that (1) all behavior (whether it is described as respondent or operant) is always interrelated with individuals’ present contexts (even when this is not apparent to observers); and (2) contexts are multifaceted, constantly evolving fields of relations between behavior, antecedent and consequent stimuli, and motivational conditions (Goldiamond, 1975; Hayes & Fryling, 2018; Kantor, 1958). In place of the prevailing respondent–operant behavior dichotomy, it may be useful for behavior analysts to think about behaviors in terms of a two-factor continuum pertaining to the extent to which behaviors (1) reliably occur in the presence of certain antecedent events and/or (2) are modifiable by consequential manipulations (see e.g., Catania, 1971). Such an approach suggests different types of treatment strategies for different behaviors along this continuum while maintaining the assumption that behavior is never independent of context, even if relations between them may be difficult to identify. This approach may contribute to the observation of previously overlooked regularities in behavior–context relations (including the consideration of the effects of historical factors that are less apparent to observers in the present; Bouton & Balleine, 2019) and the development of measurement and assessment methods that more effectively capture these (Jessel et al., 2016; Rajamaran et al., 2022). This may also contribute to a more sophisticated analysis of factors involved in the generalization and maintenance of behavior change outside of treatment settings, a prevailing challenge for the social significance of ABA treatment (Podlesnik et al., 2017; Stokes & Baer, 1978).

### Pavlovian Relations in Operant Procedures

As we noted earlier, operant procedures also involve Pavlovian relations that influence their outcomes. Recognizing these in the interpretation of behavior and the development of ABA treatment strategies may enhance their efficacy and efficiency, or contribute to the development of novel procedures.

Autoshaping and PIT research demonstrates that positive reinforcers and aversive stimuli commonly used in operant conditioning procedures, as well as the stimuli reliably associated with them, often have “eliciting” functions relevant to understanding the effects of operant conditioning procedures. The eliciting functions of unconditioned reinforcers/aversive stimuli may be considered analogous to those elicited by USs, whereas the eliciting functions of conditioned reinforcers/aversive stimuli are analogous to those elicited CSs. As we noted earlier, characterizing these responses as elicited does not imply that (1) responses to unconditioned reinforcers/aversive stimuli and conditioned reinforcers/aversive stimuli associated with them are the same; (2) they are limited to “glandular” responses only relevant to the “internal economy” of individuals; or (3) they are insensitive to context. Rather, these responses may become involved in operant procedures and either facilitate or impede the performances that behavior analysts are trying to establish and maintain. For expository purposes, these may be categorized and discussed in terms of a continuum between (1) “overt” response forms that are more readily observed by others and may have a direct effect on the external environment; and (2) “covert” response forms that are less readily observed but have effects on behavior that are often described as motivational or emotional in nature. We discuss each of these response forms next.

#### Elicitation of Overt Response Forms

Autoshaping experiments demonstrate that reinforcers elicit a variety of overt response forms, including orienting to and approaching stimuli reliably associated with their presentation (sign tracking) and/or the location where they are presented (goal tracking). Aversive stimuli also elicit species-specific overt response forms that may participate in operant escape and avoidance contingencies (Domjan, 1983; Fanselow et al., 1987; Smith et al., 1972). The response forms elicited by reinforcers/aversive stimuli depend upon the properties of the reinforcers (e.g., food versus water reinforcers; Jenkins & Harris, 1973), the contexts in which they occur (Burns & Domjan, 2001), and the temporal relationship between reinforcers/aversive stimuli and other stimuli reliably associated with them (Balsam et al., 2010). Many of these may facilitate learning brought about through operant contingencies. For example, orientation and approach to stimuli reliably associated with reinforcers may facilitate sign tracking/attention to antecedent stimuli that may acquire a degree of control over behavior (e.g., discriminative stimuli, models, or prompts). ABA procedures for autistic individuals historically involved directly reinforcing “eye contact” behavior as a means of increasing the probability that learners attend to instructional stimuli (e.g., Lovaas, 2002). This practice has become controversial, but attention to stimuli involved in instruction remains an indispensable prerequisite for social learning (Degli Espinosa, 2025). Identifying (e.g., through preference assessment) and employing effective reinforcers that elicit attention to stimuli associated with them (i.e., procedures that promote sign tracking) is an alternative strategy.

Overt response forms elicited by reinforcers or aversive stimuli and the stimuli associated with them may also impede what behavior analysts are trying to teach via operant contingencies. For example, orientation/attention to the properties of reinforcers or aversive stimuli (i.e., goal tracking) may overshadow or prevent attention to other features of the treatment context that behavior analysts may be trying to establish as discriminative stimuli. In addition, behaviors potentially elicited by aversive stimuli (often described as “emotional” in nature, including aggressive or self-injurious behaviors) may complicate ABA treatment, or in some cases be the primary reason for it. The typical approach to addressing these is based upon an operant analysis of the reinforcer(s) maintaining them (functional assessment). This approach is effective when aggressive or self-injurious behavior has been influenced by operant contingencies, but in some circumstances these behaviors may have more of an “elicited” character in the sense that they are reliably occasioned by specific aversive stimuli and may be less sensitive to modification through the use of operant contingencies (Lewon & Hayes, 2014; Lewon et al., 2019). This seems especially likely for younger individuals for whom these behaviors have shorter/less varied histories of reinforcement (Lewon et al., 2019). It has also been suggested that challenging behaviors of individuals who have experienced histories of trauma arising from unpredictable and uncontrollable aversive stimuli may be particularly sensitive to conditioned aversive “triggers” and less responsive to conventional operant conditioning treatment approaches focused on the assessment and manipulation of consequential events, or at least warrant special consideration (Ferrari et al., 2024; Jessel et al., 2024; Rajamaran et al., 2022). For these reasons, the development of treatment strategies for aggressive and self-injurious behavior would benefit from considering Pavlovian processes (e.g., graduated exposure and/or reconditioning by associating eliciting stimuli with reinforcing events).

#### Elicitation of Motivational States

Conditioned suppression and PIT studies demonstrate that presenting reinforcers or aversive stimuli, or stimuli associated with them in Pavlovian relations, can also elicit motivational states that have effects on responding under operant contingencies. As noted, superimposing conditioned aversive stimuli established through Pavlovian conditioning may reduce responding reinforced using operant contingencies, whereas superimposing CSs associated with reinforcers through Pavlovian conditioning may increase positively reinforced operant behavior. In addition, aversive stimuli and cues associated with them through Pavlovian conditioning can increase aggressive behaviors when the only consequences for these are the opportunity to attack objects or other individuals (Lewon & Hayes, 2014; Lewon et al., 2019). The effects of these stimuli, established through Pavlovian conditioning, are characterized as motivational or emotional in nature because they alter measures of behavior under operant contingencies even when the other relations in the contingencies (i.e., those between discriminative stimuli, behavior, and consequences) remain unchanged.

The implication of this for ABA is that the CSs associated with reinforcers or aversive stimuli through Pavlovian relations are a potentially important means by which behavior analysts may manage motivational and emotional conditions. Many motivational factors are outside of the control of practitioners (e.g., sleep, diet, medication, illness), but reinforcers, in addition to their “operant” function, can also elicit motivational states (Lewon & Hayes, 2014). Mood induction procedures are an example of a tool that could be used by practitioners to leverage these motivational functions of reinforcers and the stimuli associated with them. Carr et al. (2003), for example, conducted mood ratings through observable signs of affect and found that “bad moods” were predictive of severe challenging behaviors. They then developed a mood induction procedure that was implemented when mood ratings indicated bad moods. The procedure involved periodic response-independent delivery of reinforcers identified through preference assessments. No other contingency manipulations occurred. They found that the mood induction procedure both improved mood ratings and produced dramatic decreases in challenging behaviors. These types of procedures may have their effects on challenging behavior by increasing the reinforcing functions of events in the treatment setting and/or decreasing the aversive properties of other events (e.g., task demands, refusal of requests; Lewon & Hayes, 2014).

### Pavlovian Antecedent-Consequence Relations in Operant Conditioning

Pavlovian stimulus–stimulus relations are always embedded in and inseparable from the operant contingencies arranged by behavior analysts (Delgado & Hayes, 2014). Some of these are commonly acknowledged such as those involved in establishing conditioned reinforcers (Madden et al., 2023) and conditioned aversive stimuli (Dinsmoor, 2001; Nasser & McNally, 2012). However, Pavlovian relations between antecedent and consequential events (A–C Pavlovian relations) are often overlooked. When behavior analysts arrange something like a discrete-trial procedure wherein an antecedent discriminative stimulus is presented, a response occurs, and is followed by a consequence, they are arranging relations not just between the response and the consequence, but also Pavlovian relations between the discriminative stimulus and the consequence (Colwill & Rescorla, 1986). When this relationship is relatively reliable, as it often is in teaching contexts, some functions of consequential events may come to operate in part through antecedent events with which they are associated.

The main implication of this for ABA is that the nature of the A–C relations embedded in operant procedures affect the outcomes of these that are not necessarily predicted or expected by a strictly “response strengthening/weakening” view of the effects of reinforcement. As an example, research has shown that discrimination learning is enhanced when different reinforcers are used in training different discriminations. This has been described as the *differential outcomes effect* (Catania, 2025; Trapold, 1970; Urcuioli, 2005). In basic studies, animals are taught to engage in one response B1 in the presence of a discriminative stimulus A1 and to engage in a different response B2 in the presence of a different discriminative stimulus A2. B1 is reinforced when it occurs in the presence of A1 (but not in the presence of A2), and vice versa for B2. Differential outcomes research shows that animals learn this discrimination to criteria in substantially fewer trials/sessions when two different reinforcers are used (A1–B1–**C1**, A2–B2–**C2)** relative to when the same reinforcer is used for both discriminations (A1–B1–**C1**, A2–B2–**C1**).

Imagine an applied situation in which a behavior analyst is trying to teach a learner to accurately tact “surprised” versus “scared” when presented with different cards showing people’s facial expressions. The antecedent stimuli (both the shape/size of the cards and the facial expressions shown on them) share many features, which may make the discrimination difficult. The establishment of this discrimination may be facilitated by providing one reinforcer contingent upon correct “surprise” tacts and a different one contingent upon correct “scared” tacts. It is presumed that differential outcomes enhance the establishment of the discrimination because the two different responses are associated with two different sets of A–C relations (A1–C1, A2–C2, rather than just two different antecedent stimuli with the same reinforcer (A1/A2–C1). Another way of stating this is that the different properties of reinforcers C1 and C2 come to operate through A1 and A2 due to their respective A–C relations, and this facilitates discrimination by making A1 and A2 more functionally distinct. When a suitable degree of discrimination is established in this way, it seems likely that maintenance and generalization may be promoted by introducing different reinforcers (i.e., C3, C4,... CX) for correct responses to A1 and A2 such that discriminative control of the responses come to rely less on the reinforcers associated with each and more on the antecedent stimuli intended to evoke responses outside of the teaching setting.

Another area in which A–C Pavlovian relations are relevant to ABA is in the relative susceptibility of behavior to disruption due to changes in contextual conditions. Research on behavioral momentum has shown that higher rates of reinforcement for a response (which translates to more numerous/frequent Pavlovian A–C pairings) make the response more resistant to extinction and less sensitive to changes in motivational conditions (Nevin & Shahan, 2011). Likewise, research has shown that a greater number of instances of reinforcement of a response (e.g., a response reinforced 500 times versus one that has only been reinforced 100 times) decreases the extent to which it is disrupted by changes in motivational conditions (e.g., deprivation and satiation) and changes in the value of reinforcers (e.g., through food poisoning or association of the reinforcer with other aversive events; Adams, 1982; Bouton & Balleine, 2019). Responses that have been reinforced more often and are therefore less affected by changes in motivation or reinforcer value have been characterized as “habits.” These phenomena are relevant to the persistence of challenging behaviors during operant-based treatment approaches as well as to the maintenance of behavior after treatment. Acknowledgement of the role of Pavlovian A–C relations in these situations may help behavior analysts interpret behavior and plan intervention strategies aimed at explicitly managing these to address the specific needs of their consumers.

### Conditional Relations in Behavioral Assessment

Another area in which consideration of Pavlovian relations is potentially valuable to ABA is in behavioral assessment. Many behavior analysts are involved in the assessment of challenging behaviors. A major accomplishment in ABA was the development of functional assessment/analysis procedures to guide individualized treatment strategies (Peterson & Neef, 2020). In these, researchers and practitioners aim to understand the contextual conditions under which behaviors of interest occur (or fail to occur). This information is then used to arrange contexts in ways that promote desired behavior change. Direct assessments generally take two forms: descriptive assessments (DAs) that involve observing behavior in the settings of concern and collecting data on antecedent–behavior–consequence (A–B–C) relations, and functional analyses (FAs) conducted in less naturalistic settings that permit systematically manipulating A–B–C relations.

DAs are typically conducted in naturalistic settings. Because of that, DAs cannot unequivocally demonstrate contextual control of behaviors by the events of interest, and the information collected is not always easily quantifiable. FAs have been represented as the “gold standard” assessment method (Contreras et al., 2023) because they can demonstrate functional relations between behavior and context, and the information obtained from them is quantifiable. But FAs are also time and resource intensive, and are conducted in controlled settings that may not fully reflect the conditions under which behaviors occur ordinarily. The operant three-term contingency formulation is the basis for both DA and FA assessment procedures.

We have suggested that Pavlovian and operant conditioning procedures may be characterized in terms of various overlapping relations between context and behavior. Because of this, the analysis of conditional relations may be a valuable tool to incorporate into behavioral assessment in ABA. Conditional relations (sometimes described as conditional probabilities) are quantitative measures of relations between two or more events, most often calculated in terms of the occurrence of one event given the occurrence of another, along with the calculation of the occurrence of one event given the absence of the other. In the Pavlovian conditioning literature, CS–US relations are often studied in these terms (e.g., the number of times a CS occurs with an US divided by the total number of times the CS occurs with and without the CS during a period of observation). This measure provides more information about the CS–US associative relation than a simple count of the number of times the CS and US are paired. It is important to note that conditional relations between CS and US are more predictive of the outcomes of Pavlovian conditioning procedures than a simple count of the number of CS–US pairings (Rescorla, 1967, 1988).

Bijou et al. (1968) suggested that the analysis of conditional relations in ABA assessment might have value in combining the strengths of the FA and DA approaches (quantifiable data from FA with naturalistic observation from DA). Measurement of conditional relations among classes of antecedent events, behavior, and consequential events may provide valuable assessment information about relations between events that could reflect both Pavlovian and operant relations in individuals’ environments. In general, this would involve first operationally defining classes of antecedent events (As), behaviors (Bs), and consequential events (Cs). Then, observation and measurement similar to what typically occurs in A–B–C DAs would be conducted, and the classes of A and C events would be recorded upon each instance of B. These data would allow for the calculation of conditional A–B, B–C, A–C, and A–B–C relations. A–B conditional relations allow for an analysis of the regularity with which various antecedent events occasion behavior, including the identification of reliable “triggers.” B–C conditional relations provide information about the extent to which various consequential events occur after behavior, i.e., the identification of potential reinforcers. Analyses of A–C relations allow for the assessment of the Pavlovian relationship between antecedent and consequential events. A–B–C conditional relations would identify the most common *combinations* of antecedent and consequent events associated with behavior. This approach allows for a great degree of flexibility as it does not require a commitment to conceptual dichotomies between respondent versus operant behavior, USs versus reinforcers/aversive stimuli, or CSs versus discriminative stimuli. It simply involves observing and quantifying relations between classes of specified events. As such, it may be tailored to the unique naturalistic contexts of individuals receiving ABA services and allow behavior analysts to identify the features of context to which treatment strategies may be most efficiently applied. Although some ABA assessment research has investigated variations on this approach (Contreras et al., 2023; McComas et al., 2009; Pence et al., 2009), it remains understudied relative to FA approaches, and the extent to which practitioners incorporate quantitative analyses of conditional relations in DAs outside of research settings is not clear.

## Conclusion

Experimental analyses have greatly expanded our understanding of the relationships between what Skinner called respondent and operant behavior and respondent and operant conditioning. One of the views that warrants reconsideration is that Pavlovian conditioned responses (CRs) are limited to glandular responses. Many CRs established through Pavlovian conditioning, including paradigmatic responses in operant conditioning research (key pecking in pigeons and lever pressing in rats), directly affect the environment and may either facilitate or limit the outcomes that may be achieved via operant procedures. Because of this, we suggest moving past the prevailing distinction between respondent and operant behavior. Another view that could be gainfully abandoned is that operant and Pavlovian conditioning are independent forms of learning, or that it is possible to conduct an operant conditioning procedure in the absence of Pavlovian relations. A more defensible approach, and the approach we advocate, is to recognize the possible involvement of Pavlovian processes in operant procedures and figure out how to leverage this to devise more effective and efficient teaching procedures.

The failure to appreciate the important role of Pavlovian relationships in operant contingencies has prevented the translation of a modern understanding of Pavlovian conditioning into application. This unnecessarily limits the analytic tools available to applied behavior analysts. A modern view of Pavlovian conditioning and its relevance to operant procedures common in ABA would help applied behavior analysts orient to clinical issues in new ways and lead to the development of possibly more effective approaches to behavioral assessment/intervention and the maintenance and generalization of behavior change outside of treatment settings.

## References

[CR1] Adams, C. D. (1982). Variations in the sensitivity of instrumental responding to reinforcer devaluation. *Quarterly Journal of Experimental Psychology,**34*(2b), 77–98. 10.1080/14640748208400878

[CR2] Alpers, G. W. (2024). Happy 100^th^ anniversary, behavior therapy! *Behaviour Research and Therapy,**183*, 104642. 10.1016/j.brat.2024.104642 39428286 10.1016/j.brat.2024.104642

[CR3] Atnip, G. W. (1977). Stimulus- and response-reinforcer contingencies in autoshaping, operant, classical, and omission training in rats. *Journal of the Experimental Analysis of Behavior,**28*, 59–69. 10.1901/jeab.1977.28-59 16812014 10.1901/jeab.1977.28-59 PMC1333614

[CR4] Baer, D. M., Wolf, M. M., & Risley, T. R. (1968). Some current dimensions of applied behavior analysis. *Journal of Applied Behavior Analysis,**1*(1), 91–97. 10.1901/jaba.1968.1-91 16795165 10.1901/jaba.1968.1-91 PMC1310980

[CR5] Badioli, M., Degni, L. A. E., Dalbagno, D., Danti, C., Starita, F., di Pellegrino, G., Benassi, M., & Garofalo, S. (2024). Unraveling the influence of Pavlovian cues on decision-making: A pre-registered meta-analysis on Pavlovian-to-instrumental transfer. *Neuroscience and Biobehavioral Reviews,**164*, 105829. 10.1016/j.neubiorev.2024.105829 39074674 10.1016/j.neubiorev.2024.105829

[CR6] Balsam, P. D., Drew, M. R., & Gallistel, C. R. (2010). Time and associative learning. *Comparative Cognition and Behavior Reviews,**5*, 1–22. 10.3819/ccbr.2010.50001 21359131 10.3819/ccbr.2010.50001 PMC3045055

[CR7] Barlow, D. H. (1997). Promises to keep. *Behavior Therapy,**28*(4), 589–596. 10.1016/S0005-7894(97)80017-2

[CR8] Behavior Analyst Certification Board. (2012). *Task list* (4^th^ ed.).

[CR9] Behavior Analyst Certification Board. (2017). *BCBA/BCaBA task list* (5^th^ ed.).

[CR10] Behavior Analyst Certification Board. (2022). *BCBA test content outline* (6^th^ ed.).

[CR11] Behavior Analyst Certification Board. (2024). *Board certified behavior analyst handbook*.

[CR12] Benjamin, L. T. (2005). A history of clinical psychology as a profession in America (and a glimpse at its future). *Annual Review of Clinical Psychology,**1*, 1–30. 10.1146/annurev.clinpsy.1.102803.143758 17716080 10.1146/annurev.clinpsy.1.102803.143758

[CR13] Bijou, S. W., Peterson, R. F., & Ault, M. H. (1968). A method to integrate descriptive and experimental field studies at the level of data and empirical concepts. *Journal of Applied Behavior Analysis,**1*(2), 175–191. 10.1901/jaba.1968.1-175 16795175 10.1901/jaba.1968.1-175 PMC1310995

[CR14] Blackman, D. (1977/2022). Conditioned suppression and the effects of classical conditioning on operant behavior. In W. K. Honig & J. E. R. Staddon (Eds.), *Handbook of operant behavior* (pp. 340–363). Prentice-Hall.

[CR15] Bouton, M. E., & Swartzentruber, D. (1991). Sources of relapse after extinction in Pavlovian and instrumental learning. *Clinical Psychology Review,**11*(2), 123–140. 10.1016/0272-7358(91)90091-8

[CR16] Bouton, M. E., & Balleine, B. W. (2019). Prediction and control of operant behavior: What you see is not all there is. *Behavior Analysis: Research and Practice,**19*(2), 202–212. 10.1037/bar0000108 31588411 10.1037/bar0000108 PMC6777851

[CR17] Brown, P. L., & Jenkins, H. M. (1968). Auto-shaping of the pigeon’s key peck. *Journal of the Experimental Analysis of Behavior,**11*, 1–8. 10.1901/jeab.1968.11-1 5636851 10.1901/jeab.1968.11-1 PMC1338436

[CR18] Burns, M., & Domjan, M. (2001). Topography of spatially directed conditioned responding: Effects of context and trial duration. *Journal of Experimental Psychology: Animal Behavior Processes,**27*(3), 269. 11497325

[CR19] Cain, C. K., & LeDoux, J. E. (2007). Escape from fear: A detailed behavioral analysis of two atypical responses reinforced by CS termination. *Journal of Experimental Psychology: Animal Behavior Processes,**33*(4), 451–463. 10.1037/0097-7403.33.4.451 17924792 10.1037/0097-7403.33.4.451

[CR20] Campolattaro, M. M., Schnitker, K. M., & Freeman, J. H. (2008). Changes in inhibition during differential eyeblink conditioning with increased training. *Learning & Behavior,**36*, 159–165. 10.3758/LB.36.2.159 18543716 10.3758/lb.36.2.159 PMC2556363

[CR21] Carr, E. G., McLaughlin, D. M., Giacobbe-Grieco, T., & Smith, C. E. (2003). Using mood ratings and mood induction in assessment and intervention for severe problem behavior. *American Journal on Mental Retardation,**108*(1), 32–55. 10.1352/0895-8017(2003)108<0032:UMRAMI>2.0.CO;2 12475365 10.1352/0895-8017(2003)108<0032:UMRAMI>2.0.CO;2

[CR22] Cartoni, E., Balleine, B., & Baldassarre, G. (2016). Appetitive pavlovian-instrumental transfer: A review. *Neuroscience and Biobehavioral Reviews,**71*, 829–848. 27693227 10.1016/j.neubiorev.2016.09.020

[CR23] Catania, A. C. (1971). Elicitation, reinforcement, and stimulus control. In *The nature of reinforcement* (pp. 196–220). Academic Press.

[CR24] Catania, A. C. (2008). The *journal of the experimental analysis of behavior* at fifty and one hundred. *Journal of the Experimental Analysis of Behavior,**89*(1), 111–118. 10.1901/jeab.2008.89-111 18338678 10.1901/jeab.2008.89-111 PMC2211432

[CR25] Catania, A. C. (2025). Urcuioli’s differential-outcomes research: Implications for our behavioral units. *Journal of the Experimental Analysis of Behavior,**124*(1), e700018. 10.1002/jeab.70018 10.1002/jeab.70018 40266649

[CR26] Chance, P. (1999). Thorndike’s puzzle boxes and the origins of the experimental analysis of behavior. *Journal of the Experimental Analysis of Behavior,**72*, 433–440. 10.1901/jeab.1999.72-433 16812921 10.1901/jeab.1999.72-433 PMC1284753

[CR27] Cleland, G. G., & Davey, G. C. L. (1983). Autoshaping in the rat: The effects of localizable visual and auditory signals for food. *Journal of the Experimental Analysis of Behavior,**40*, 47–56. 10.1901/jeab.1983.40-47 16812336 10.1901/jeab.1983.40-47 PMC1347843

[CR28] Colaizzi, J. M., Flagel, S. B., Joyner, M. A., Gearhardt, A. N., Stewart, J. L., & Paulus, M. P. (2020). Mapping sign-tracking and goal-tracking onto human behaviors. *Neuroscience and Biobehavioral RevieWs,**111*, 84–94. 31972203 10.1016/j.neubiorev.2020.01.018 PMC8087151

[CR29] Colwill, R. M., & Rescorla, R. A. (1986). Associative structures in instrumental learning. *Psychology of Learning & Motivation,**20*, 55–104. 10.1016/S0079-7421(08)60016-X

[CR30] Contreras, B. P., Tate, S. A., Morris, S. L., & Kahng, S. (2023). A systematic review of the correspondence between descriptive assessment and functional analysis. *Journal of Applied Behavior Analysis,**56*(1), 146–165. 10.1002/jaba.958 36409837 10.1002/jaba.958

[CR31] Cooper, J. O., Heron, T. E., & Heward, W. L. (2020). *Applied behavior analysis* (3rd ed.). Pearson.

[CR32] Corbit, L. H., & Balleine, B. W. (2016). Learning and motivational processes contributing to Pavlovian-instrumental transfer and their neural bases: Dopamine and beyond. *Current Topics in Behavior Neuroscience,**27*, 259–289. 10.1007/7854_2015_388 10.1007/7854_2015_388 26695169

[CR33] Degli Espinosa, F. (2025). Eye contact: To teach or not to teach? That is the not the question. *Perspectives on Behavior Science,**48*, 529–537. 10.1007/s40614-025-00456-2 40919353 10.1007/s40614-025-00456-2 PMC12411364

[CR34] Degni, L. A. E., Dalbagno, D., Starita, F., Benassi, M., di Pellegrino, G., & Garofalo, S. (2022). General Pavlovian-to-instrumental transfer in humans: Evidence from Bayesian inference. *Frontiers in Behavioral Neuroscience,**16*, 945503. 10.3389/fnbeh.2022.945503 36051636 10.3389/fnbeh.2022.945503 PMC9426756

[CR35] Delgado, D., & Hayes, L. J. (2014). An integrative approach to learning processes: Revisiting substitution of functions. *The Psychological Record,**64*, 625–637. 10.1007/s40732-014-0071-6

[CR36] Dinsmoor, J. A. (1995). Stimulus control: Part I. *The Behavior Analyst,**18*(1), 51–68. 10.1007/BF03392691 22478204 10.1007/BF03392691 PMC2733673

[CR37] Dinsmoor, J. A. (2001). Stimuli inevitably generated by behavior that avoids electric shock are inherently reinforcing. *Journal of the Experimental Analysis of Behavior,**75*(3), 311–333. 10.1901/jeab.2001.75-311 11453621 10.1901/jeab.2001.75-311 PMC1284820

[CR38] Dinsmoor, J. A., Flint, G. A., Smith, R. F., & Viemeister, N. F. (1969). Differential reinforcing effects of stimuli associated with the presence or absence of a schedule of punishment. In D. P. Hendry (Ed.), *Conditioned reinforcement* (pp. 357–384). Dorsey.

[CR39] Domjan, M. (1983). Biological constraints on instrumental and classical conditioning. *Psychology of Learning and Motivation,**17*, 215–277. 10.1016/S0079-7421(08)60100-0

[CR40] Domjan, M. (2016). Elicited versus emitted behavior: Time to abandon the distinction. *Journal of the Experimental Analysis of Behavior,**105*, 231–245. 10.1002/jeab.197 26872681 10.1002/jeab.197

[CR41] Domjan, M. (2026). Thorndike’s law of effect and its inconsistent description over the years. *Journal of the Experimental Analysis of Behavior,**125*(1), e70073. 10.1002/jeab.70073 41363971 10.1002/jeab.70073 PMC12687759

[CR42] Donohoe, J. W., & Vegas, R. (2021). Respondent (Pavlovian) conditioning. In W. W. Fischer, C. C. Piazza, & H.S. Roane (Eds.), *Handbook of applied behavior analysis* (2nd ed., pp. 15–36). The Guilford Press.

[CR43] Ellison, G. D., & Konorski, J. (1964). Separation of the salivary and motor responses in instrumental conditioning. *Science,**146*(3647), 1071–1072. 10.1126/science.146.3647.1071 14202465 10.1126/science.146.3647.1071

[CR44] Estes, W. K., & Skinner, B. F. (1941). Some quantitative properties of anxiety. *Journal of Experimental Psychology,**29*, 390–400. 10.1037/h0062283

[CR45] Estes, W. K. (1948). Discriminative conditioning. II. Effects of a Pavlovian conditioned stimulus upon a subsequently established operant response. *Journal of Experimental Psychology,**38*, 173–177. 10.1037/h0057525 18913666 10.1037/h0057525

[CR46] Fanselow, M. S., Sigmundi, R. A., & Williams, J. L. (1987). Response selection and the hierarchical organization of species-specific defense reactions: The relationship between freezing, flight, and defensive burying. *The Psychological Record,**37*, 381–386.

[CR47] Ferrari, C., Lewon, M., & Falletta-Cowden, N. (2024). Structured peer observation and targeted training to increase consistency of classroom practices in a self-contained school for students with emotional and behavioral disorders. *Journal of Educational & Psychological Consultation,**35*(1), 88–113. 10.1080/10474412.2024.2352471

[CR48] Ferster, C. B., & Skinner, B. F. (1957). *Schedules of reinforcement*. Appleton-Century-Crofts.

[CR49] Flagel, S. B., Akil, H., & Robinson, T. E. (2009). Individual differences in the attribution of incentive salience to reward-related cues: Implications for addiction. *Neuropharmacology,**56*(Supplement 1), 139–148. 10.1016/j.neuropharm.2008.06.027 18619474 10.1016/j.neuropharm.2008.06.027 PMC2635343

[CR50] Goldiamond, I. (1975). Alternative sets as a framework for behavioral formulations and research. *Behaviorism,**3*(1), 49–86.

[CR51] Hayes, L. J., & Fryling, M. J. (2018). Psychological events as integrated fields. *The Psychological Record,**68*, 273–277. 10.1007/s40732-018-0274-3

[CR52] Hayes, L. J., & Fryling, M. J. (2019). Functional and descriptive contextualism. *Journal of Contextual Behavior Science,**14*, 119–126. 10.1016/j.jcbs.2019.09.002

[CR53] Hayes, S. C., Hayes, L. J., & Reese, H. W. (1988). Finding the philosophical core: A review of Stephen C. Pepper’s *World hypotheses: A study in evidence. Journal of the Experimental Analysis of Behavior, 50*(1), 97–111. 10.1901/jeab.1988.50-97 10.1901/jeab.1988.50-97 PMC1338844 16812552

[CR54] Hayes, S. C., Strosahl, K. D., & Wilson, K. G. (2012). *Acceptance and commitment therapy: The process and practice of mindful change* (2^nd^ ed.)*.* Guilford Press.

[CR55] Hershberger, W. A. (1986). An approach through the looking-glass. *Animal Learning & Behavior,**14*(4), 443–451. 10.3758/BF03200092

[CR56] Holland, P. C. (2004). Relations between pavlovian-instrumental transfer and reinforcer devaluation. *Journal of Experimental Psychology: Animal Behavior Processes,**30*(2), 104–117. 15078120 10.1037/0097-7403.30.2.104

[CR57] Holmes, N. M., Marchand, A. R., & Coutureau, E. (2010). Pavlovian to instrumental transfer: A neurobehavioural perspective. *Neuroscience and Biobehavioral RevieWs,**34*(8), 1277–1295. 10.1016/j.neubiorev.2010.03.007 20385164 10.1016/j.neubiorev.2010.03.007

[CR58] Honig, W. K., & Urcuioli, P. J. (1981). The legacy of Guttman and Kalish (1956): 25 years of research on stimulus generalization. *Journal of the Experimental Analysis of Behavior,**36*(3), 405–445. 10.1901/jeab.1981.36-405 16812256 10.1901/jeab.1981.36-405 PMC1333109

[CR59] Hull, C. L. (1930). Goal attraction and directing ideas conceived as habit phenomena. *Psychological Review,**38*, 487–506.

[CR60] Hull, C. L. (1931). Knowledge and purpose as habit mechanisms. *Psychological Review,**30*, 511–525.

[CR61] Jenkins, H. M., Barrera, F. J., Ireland, C., & Woodside, B. (1978). Signal-centered action patterns of dogs in appetitive classical conditioning. *Learning and Motivation, 9*(3), 272–296. 10.1016/0023-9690(78)90010-3

[CR62] Jessel, J., Fruchtman, T., Raghunauth-Zaman, N., Leyman, A., Lemos, F. M., Val, H, C., Howard, M., & Hanley, G. P. (2024). A two step validation of the performance-based IISCA: A trauma-informed functional analysis model. *Behavior Analysis in Practice, 17*, 727–745. 10.1007/s40617-023-00792-2 10.1007/s40617-023-00792-2 PMC11461361 39391176

[CR63] Jessel, J., Hanley, G. P., & Ghaemmaghami, M. (2016). Interview-informed synthesized contingency analyses: Thirty replications and reanalysis. *Journal of Applied Behavior Analysis,**49*(3), 576–595. 10.1002/jaba.316 27174653 10.1002/jaba.316

[CR64] Jones, M. C. (1924a). The elimination of children’s fears. *Journal of Experimental Psychology,**7*, 382–390. 10.1037/h0072283

[CR65] Jones, M. C. (1924b). A laboratory study of fear: The case of Peter. *The Pedagogical Seminary and Journal of Genetic Psychology,**31*, 308–315. 10.1080/00221325.1991.9914707 1817149

[CR66] James, W. (1907). Pragmatism, a new name for some old ways of thinking*.* In *Popular lectures on philosophy* (pp. 3–42)*.* Longmans, Green, & Company.

[CR67] Kamin, L. J., Brimer, C. J., & Black, A. H. (1963). Conditioned suppression as a monitor of fear of the CS in the course of avoidance training. *Journal of Comparative and Physiological Psychology,**56*(3), 497–501. 10.1037/h0047966 13962088 10.1037/h0047966

[CR68] Kantor, J. R. (1958). *Interbehavioral psychology.* Principia.

[CR69] Keller, F. S., & Schoenfeld, W. N. (1950). *Principles of psychology*. Appleton-Century-Crofts.

[CR70] Köksal, F., Kumru, G., Anarat, C., Domjan, M., & Yeniçer, N. (2021). Compulsive conditioned sexual responding of male Japanese quail in extinction. *Archives of Sexual Behavior, 50*, 1207–1216. 10.1007/s10508-020-01906-5 10.1007/s10508-020-01906-5 33398697

[CR71] Krank, M. D., O’Neil, S. K., & Jacobs, J. (2008). Goal- and signal-directed incentive: Conditioned approach, seeking, and consumption established with unsweetened alcohol in rats. *Psychopharmacology (Berl),**196*, 397–405. 17965977 10.1007/s00213-007-0971-0

[CR72] Kruse, J. M., Overmier, J. B., Konz, W. A., & Rokke, E. (1983). Pavlovian conditioned stimulus effects upon instrumental choice behavior are reinforcer specific. *Learning & Motivation,**14*, 165–181.

[CR73] Leão, M., da Rocha, C. A. A., & Laurenti, C. (2016). A reassessment of pragmatism in behavior analysis: I. The theory of truth. *Revista Mexicana de Análisis de la Conducta, 42*(1), 87–104.

[CR74] LeDoux, J. E., Moscarello, J., Sears, R., & Campese, V. (2017). The birth, death and resurrection of avoidance: A reconceptualization of a troubled paradigm. *Molecular Psychiatry,**22*(1), 24–36. 27752080 10.1038/mp.2016.166 PMC5173426

[CR75] Lewon, M., & Hayes, L. J. (2014). Toward an analysis of emotions as products of motivating operations. *The Psychological Record,**64*, 813–825. 10.1007/s40732-014-0046-7

[CR76] Lewon, M., Houmanfar, R. A., & Hayes, L. J. (2019). The will to fight: Aversion-induced aggression and the role of motivation in intergroup conflict. *Perspectives on Behavior Science,**42*, 889–910. 10.1007/s40614-019-00221-2 31976465 10.1007/s40614-019-00221-2 PMC6901646

[CR77] Lovaas, O. I. (2002). *Teaching individuals with developmental delays: Basic intervention techniques*. Pro Ed.

[CR78] Lyons, D. O. (1968). Conditioned suppression: Operant variables and aversive control. *Psychological Record,**18*, 317–338. 10.1007/BF03393779

[CR79] Madden, G. J., Mahmoudi, S., & Brown, K. (2023). Pavlovian conditioning and conditioned reinforcement. *Journal of Applied Behavior Analysis,**56*(3), 498–519. 10.1002/jaba.1004 37254881 10.1002/jaba.1004 PMC10364091

[CR80] Mahlberg, J., Seabrooke, T., Weidemann, G., Hogarth, L., Mitchell, C. J., & Moustafa, A. A. (2021). Human appetitive Pavlovian-to-instrumental transfer: A goal-directed account. *Psychological Research Psychologische Forschung,**85*, 449–463. 10.1007/s00426-019-01266-3 31720789 10.1007/s00426-019-01266-3

[CR81] Malott, R. W., & Kohler, K. T. (2021). *Principles of behavior* (8^th^ ed.). Routledge.

[CR82] María-Ríos, C. E., Fitzpatrick, C. J., Czesak, F., & Morrow, J. D. (2023). Effects of predictive and incentive value manipulation on sign- and goal-tracking behavior. *Neurobiology of Learning and Memory,**203*, 107796. 10.1016/j.nlm.2023.107796 37385521 10.1016/j.nlm.2023.107796 PMC10599606

[CR83] Martin, G., & Pear, J. (2019). *Behavior modification: What it is and how to do it* (11^th^ ed.). Routledge.

[CR84] McComas, J. J., Moore, T., Dahl, N., Hartman, E., Hoch, J., & Symons, F. (2009). Calculating contingencies in natural environments: Issues in the application of sequential analysis. *Journal of Applied Behavior Analysis,**42*(2), 413–423. 10.1901/jaba.2009.42-413 19949534 10.1901/jaba.2009.42-413 PMC2695351

[CR85] Michael, J. (1980). Flight from behavior analysis. *The Behavior Analyst,**3*(2), 1–21. 10.1007/BF03391838 22478477 10.1007/BF03391838 PMC2741836

[CR86] Michael, J. (2004). *Concepts and principles of behavior analysis*. Association for Behavior Analysis International.

[CR87] Mineka, S. (1979). The role of fear in theories of avoidance learning, flooding, and extinction. *Psychological Bulletin,**86*(5), 985–1010. 10.1037/0033-2909.86.5.985

[CR88] Morris, E. K., Smith, N. G., & Altus, D. E. (2005). B. F. Skinner’s contributions to applied behavior analysis. *The Behavior Analyst,**28*, 99–131. 10.1007/BF03392108 22478444 10.1007/BF03392108 PMC2755377

[CR89] Mowrer, O. H. (1947). On the dual nature of learning: A reinterpretation of “conditioning” and “problem-solving.” *Harvard Educational Review,**17*, 102–148.

[CR90] Nasser, H. M., & McNally, G. P. (2012). Appetitive-aversive interactions in Pavlovian fear conditioning. *Behavioral Neuroscience,**126*(3), 404–422. 10.1037/a0028341 22642885 10.1037/a0028341

[CR91] Nevin, J. A., & Shahan, T. A. (2011). Behavioral momentum theory: Equations and applications. *Journal of Applied Behavior Analysis,**44*(4), 877–895. 10.1901/jaba.2011.44-877 22219536 10.1901/jaba.2011.44-877 PMC3251288

[CR92] Overmier, J. B., & Lawry, J. A. (1979). Pavlovian conditioning and the mediation of behavior. *Psychology of Learning & Motivation,**13*, 1–55. 10.1016/S0079-7421(08)60080-8

[CR93] Pavlov, I. P. (1906). The scientific investigation of the psychical faculties or processes in the higher animals. *Science,**24*, 613–619. 10.1126/science.24.620.613 17771162 10.1126/science.24.620.613

[CR94] Pavlov, I. P. (1932). The reply of a physiologist to psychologists. *Psychological Review,**39*(2), 91–127. 10.1037/h0069929

[CR95] Pence, S. T., Roscoe, E. M., Bourret, J. C., & Ahearn, W. H. (2009). Relative contributions of three descriptive methods: Implications for behavioral assessment. *Journal of Applied Behavior Analysis,**42*(2), 425–446. 10.1901/jaba.2009.42-425 19949536 10.1901/jaba.2009.42-425 PMC2695353

[CR96] Peterson, G. B., Ackil, J. E., Frommer, G. P., & Hearst, E. (1972). Conditioned approach and contact behavior toward signals for food or brain stimulation reinforcement. *Science,**177*, 1009–1011. 17788815 10.1126/science.177.4053.1009

[CR97] Peterson, S. M., & Neef, N. A. (2020). Functional behavior assessment. In J. O. Cooper, T. E. Heron, & W. L. Heward (Eds.), *Applied behavior analysis* (3^rd^ ed, pp. 627–654). Pearson.

[CR98] Podlesnik, C. A., Kelley, M. E., Jimenez-Gomez, C., & Bouton, M. E. (2017). Renewed behavior produced by context change and its implications for treatment maintenance: A review. *Journal of Applied Behavior Analysis,**50*(3), 675–697. 10.1002/jaba.400 28608584 10.1002/jaba.400 PMC5538309

[CR99] Rajamaran, A., Austin, J. L., Gover, H. C., Cammilleri, A. P., Donnelly, D. R., & Hanley, G. P. (2022). *Journal of Applied Behavior Analysis, 55*(1), 40–61.10.1002/jaba.881 10.1002/jaba.881 34525220

[CR100] Rescorla, R. A. (1967). Pavlovian conditioning and its proper control procedures. *Psychological Review,**74*(1), 71–80. 10.1037/h0024109 5341445 10.1037/h0024109

[CR101] Rescorla, R. A. (1988). Pavlovian conditioning: It’s not what you think it is. *American Psychologist,**43*(3), 151–160. 10.1037/0003-066X.43.3.151 3364852 10.1037//0003-066x.43.3.151

[CR102] Rescorla, R. A., & Solomon, R. L. (1967). Two-process learning theory: Relationships between Pavlovian conditioning and instrumental learning. *Psychological Review,**74*, 151–182. 5342881 10.1037/h0024475

[CR103] Rosas, J. M., Todd, T. P., & Bouton, M. E. (2013). Context change and associative learning. *Wiley Interdisciplinary Reviews. Cognitive Science,**4*(3), 237–244. 10.1002/wcs.1225 23772263 10.1002/wcs.1225 PMC3680141

[CR104] Schwartz, B., & Gamzu, E. (2022). Pavlovian control of operant behavior: An analysis of autoshaping and its implications for operant conditioning. In W. K. Honig & J. E. R. Staddon (Eds.), *Handbook of operant behavior* (pp. 53–97). Routledge. (Original work published 1977)

[CR105] Sheffield, F. D. (1965). Relation between classical conditioning and instrumental learning. In W. F. Prokasy (Ed.), *Classical conditioning* (pp. 302–322). Appleton-Century-Crofts.

[CR106] Skinner, B. F. (1935). Two types of conditioned reflex and a pseudo type. *Journal of General Psychology,**12*(1), 66–77. 10.1080/00221309.1935.9920088

[CR107] Skinner, B. F. (1937). Two types of conditioned reflex: A reply to Konorski and Miller. *Journal of General Psychology,**16*(1), 272–279. 10.1080/00221309.1937.9917951

[CR108] Skinner, B. F. (1938). *The behavior of organisms: An experimental analysis*. Appleton-Century-Crofts.

[CR109] Skinner, B. F. (1953). *Science and human behavior*. Macmillan.

[CR110] Skinner, B. F. (1969). *Contingencies of reinforcement: A theoretical analysis*. Appleton-Century-Crofts.

[CR111] Skinner, B. F. (1971). *Beyond freedom & dignity.* Hackett.

[CR112] Smith, N. W. (2007). Events and constructs. *The Psychological Record,**57*, 169–186. 10.1007/BF03395570

[CR113] Smith, R. F., Gustavson, C. R., & Gregor, G. L. (1972). Incompatibility between the pigeons’ unconditioned response to shock and the conditioned key peck response. *Journal of the Experimental Analysis of Behavior,**18*, 147–153. 10.1901/jeab.1972.18-147 16811612 10.1901/jeab.1972.18-147 PMC1333994

[CR114] Spence, K. W. (1956). *Behavior theory and conditioning*. Yale University Press.

[CR115] Staddon, J. E. R., & Simmelhag, V. L. (1971). The “superstition” experiment: A reexamination of its implications for the principles of adaptive behavior. *Psychological Review, 78*(1), 3–43. 10.1037/h0030305

[CR116] Stokes, T. F., & Baer, D. M. (1978). An implicit technology of generalization. *Journal of Applied Behavior Analysis,**10*(2), 349–367. 10.1901/jaba.1977.10-349 10.1901/jaba.1977.10-349 PMC1311194 16795561

[CR117] Thalmayer, A. G., Toscanelli, C., & Arnett, J. J. (2021). The neglected 95% revisited: Is American psychology becoming less American? *American Psychologist,**76*(1), 116–129. 10.1037/amp0000622 32271027 10.1037/amp0000622

[CR118] Thomas, G., & O’Callaghan, M. (1981). Pavlovian principles and behaviour therapy. In G. Davey (Ed.), *Applications of conditioning theory* (pp. 129–149). Routledge.

[CR119] Thorndike, E. L. (1911/2000). *Animal intelligence*. Transaction Publishers.

[CR120] Timberlake, W., & Grant, D. L. (1975). Auto-shaping in rats to the presentation of another rat predicting food. *Science,**190*(4215), 690–692. 10.1126/science.190.4215.690

[CR121] Timberlake, W., Wahl, G., & King, D. A. (1982). Stimulus and response contingencies in the misbehavior of rats. *Journal of Experimental Psychology: Animal Behavior Processes, 8*(1), 62–85. 10.1037/0097-7403.8.1.62 7057145

[CR122] Trapold, M. A. (1970). Are expectancies based upon different positive reinforcing events discriminably different? *Learning & Motivation,**1*(2), 129–140. 10.1016/0023-9690(70)90079-2

[CR123] Trapold, M. A., & Overmier, J. B. (1972). The second learning process in instrumental conditioning. In A. H. Black & W. F. Prokasy (Eds.), *Classical conditioning II: Current theory and research* (pp. 427–452). Appleton-Century-Crofts.

[CR124] Twitmyer, E. B. (1974). A study of the knee jerk. *Journal of Experimental Psychology*, *103,* 1047–1066. (This is a republication of Twitmyer’s 1902 Ph.D. thesis at the University of Pennsylvania.) 10.1037/h0037386 4457582

[CR125] Urcuioli, P. J. (2005). Behavioral and associative effects of differential outcomes in discrimination learning. *Learning & Behavior,**33*, 1–21. 10.3758/BF03196047 15971490 10.3758/bf03196047

[CR126] Urcelay, G. P., & Domjan, M. (2022). Pavlovian conditioning. In S. Della Sala (Ed.), *Encyclopedia of behavioural neuroscience* (2nd ed., pp. 109–117). Academic Press.

[CR127] Watson, J. B. (1913). Psychology as the behaviorist views it. *Psychological Review,**20*, 158–177. 10.1037/h0074428

[CR128] Watson, J. B., & Rayner, R. (1920). Conditioned emotional reactions. *Journal of Experimental Psychology,**3*(60), 1–14. 10.1037/h0069608

[CR129] Williams, B. A. (1987). The other psychology of animal learning: A review of Mackintosh’s *Conditioning and Associative Learning*. *Journal of the Experimental Analysis of Behavior,**48*, 175–186. 10.1901/jeab.1987.48-175

[CR130] Williams, D. R., & Williams, H. (1969). Auto-maintenance in the pigeon: Sustained pecking despite contingent non-reinforcement. *Journal of the Experimental Analysis of Behavior,**12*(4), 511–520. 10.1901/jeab.1969.12-511 16811370 10.1901/jeab.1969.12-511 PMC1338642

[CR131] Williams, D. R. (1965). Classical conditioning and incentive motivation. In W. F. Prokasy (Ed.), *Classical conditioning* (pp. )*.* Appleton-Century-Crofts.

[CR132] Wolpe, J. (1958). *Psychotherapy by reciprocal inhibition*. Stanford University Press.

[CR133] Yerkes, R. M., & Morgulis, S. (1909). The method of Pawlow in animal psychology. *Psychological Bulletin,**6*(8), 257–273. 10.1037/h0070886

